# Efficacy of Different Regimens of 980 nm Low-Level Laser Therapy to Reduce Pain Caused by Elastomeric Separator Placement in Adults: A Randomized, Double-Blind, Split-Mouth Placebo-Controlled Study

**DOI:** 10.3390/jcm15103731

**Published:** 2026-05-12

**Authors:** Alireza Khandan Dezfully, Márió Gajdács, Aliz Eperke Pató, Krisztina Kárpáti, Melinda Madléna

**Affiliations:** 1 Department of Orthodontics and Pediatric Dentistry, Faculty of Dentistry, University of Szeged, Tisza Lajos krt. 64-66, 6720 Szeged, Hungary; khandan.dezfully.alireza@szte.hu (A.K.D.); eperke1998@gmail.com (A.E.P.); karpati.krisztina.margit@szte.hu (K.K.); 2 Doctoral School of Clinical Medicine, University of Szeged, 6720 Szeged, Hungary; 3 Department of Oral Biology and Experimental Dental Research, Faculty of Dentistry, University of Szeged, Tisza Lajos krt. 64-66, 6720 Szeged, Hungary; gajdacs.mario@med.u-szeged.hu; 4 Department of Public Health, Albert Szent-Györgyi Medical School, University of Szeged, Dóm tér 10, 6720 Szeged, Hungary

**Keywords:** orthodontics, procedural, low-level laser therapy, diode laser, oral health, pain experience

## Abstract

**Background:** Effective pain management is crucial during orthodontic treatments with fixed appliances, to ensure adequate patient compliance and to avoid treatment discontinuation. Photobiomodulation approaches, including Low Level Laser Therapy (LLLT) has received substantial attention, due to its potential as an effective, non-pharmacological analgesic modality, however, evidence pertaining to its efficacy is controversial. Our present study aims to evaluate the efficacy of LLLT vs. placebo, following placement of orthodontic elastic separators (ESs) in adult patients treated with fixed orthodontic appliances. **Methods:** A randomized, double-blind, split-mouth study was carried out, where *n* = 31 volunteers (18 male and 13 female; aged between 19 and 32 years) were enrolled. ESs were placed at the mesial and distal surfaces of the first permanent molars in both quadrants of lower, as well as upper jaws. Based on the assigned intervention, the four quadrants were divided as follows: three quadrants received LLLT treatment—using a 980 nm wavelength GaAlAs diode laser, with 0.1–0.2 W—according to three treatment regimes, i.e., regime 1 (R1): 6 J, continuous mode, R2: 12 J, continuous mode, and R3: 6 J, pulsed mode; while placebo treatment (P) was applied in the fourth quadrant. A questionnaire with a visual analogue scale (VAS; 0–100) was used for pain assessment (spontaneous pain and pain on mastication), scored directly after separation and after 6, 24, 48 and 72 h of both laser and placebo treatment application. **Results:** After the 24 h mark, significant differences were detected between the pain readings of LLLT-treated and placebo quadrants, both in resting position and during mastication (*p* < 0.05); pain readings were highest for R2, P, while lowest for R3 quadrants in resting position, and at R1 during mastication, respectively. **Conclusions:** Although findings of our study are exploratory in nature, they suggest that a single application of LLLT might be effective in reducing pain caused by ES placement, especially in the vulnerable 24 h following separation. Trial registration: clinicaltrials.gov ID NCT07456709 (date of registration: 2 March 2026, retrospectively registered).

## 1. Introduction

Effective reduction of pain is crucial during a variety of dental treatments—including orthodontic therapy, especially during treatments with fixed appliances—to ensure adequate compliance of patients, leading to the best possible clinical outcomes [1,2]. Existing evidence has highlighted that over 9 out of 10 patients reported experiencing substantial pain during orthodontic treatment, with as much as 30% opting to discontinue therapy [3,4,5]. In practice, before the initiation of effective orthodontic interventions, orthodontists place elastomeric separators (ESs) to make enough room for the subsequent use of orthodontic bands. This intervention may result in considerable levels of pain for the patients [6,7,8,9]. In recent years, approaches based on photobiomodulation, including Low Level Laser Therapy (LLLT), a non-surgical, minimally invasive method, have received substantial attention in a variety of dental domains [10,11], leading to improved oral health-related quality of life (OHRQoL) [12]. The clinical benefits associated with the use of LLLT has been described in temporomandibular disorders [13], orofacial pain disorders [14], xerostomia [15], reducing tooth sensitivity [16] or the recurrence of oral ulcers [17], enhancing osseointegration/implant stability [18,19], post-operative wound healing and tissue regeneration [20], tooth movement [21], and as an effective anti-inflammatory and analgesic treatment modality [22,23]. LLLT, as defined by devices operating with an output below 0.5 W, has a low energy output to preclude an increase in temperature above normal body temperature in target tissues [23]. If used judiciously, under the supervision of an experienced dentist (operator), LLLTs may present with substantial advantages [23,24,25,26] over conventional analgesic modalities in dentistry (e.g., pharmaceuticals, such as nonsteroidal anti-inflammatory drugs (NSAIDs)), as they do not present with adverse systemic effects (e.g., altering bone resorption processes, xerostomia) or contraindications associated with their use, leading to its superiority in the domain of safety [26].

Several studies—including high-quality randomized clinical trials (RCTs)—have aimed to evaluate the efficacy of LLLT in reducing pain caused by the introduction of orthodontic ESs in different contexts, either against placebo or NSAID treatments, respectively [27]. While the efficacy of LLLT over placebo has been supported by numerous RCTs and meta-analyses—within the context of ES and/or archwire placement [28,29,30,31,32,33,34,35] (although others were unable to reliably confirm it [36,37,38,39])—the comparison between LLLT and NSAIDSs is often controversial, where some studies describe the superiority of LLLT [30,40], while others describe the efficacy of LLLT as modest compared to NSAIDs [27]. Nonetheless, all respective systematic studies have highlighted that at present, due to the biases associated with the RCTs included and the poor quality of evidence, more studies are necessary to determine whether LLLT was effective in relieving orthodontic pain [27,30,32]. As LLLT currently sits at a contentious position, additional research should clarify the ideal conditions of its use (i.e. time, intensity, laser operation mode, specific indications, and patient populations) in everyday settings [22,23,41,42,43,44,45,46].

In our previous study, we assessed the efficacy of LLLT—in the form of a 980 nm wavelength, 0.1 W energy diode laser—as a modality for pain reduction following orthodontic separation in adult individuals treated with fixed orthodontic appliances, within the framework of a double-blind, placebo-controlled split-mouth study design [47]. Our earlier findings demonstrated that a continuously applied, single dose of LLLT with 6 J energy may have beneficial effects on orthodontic pain caused by ES, especially after 6 h of ES placement, achieving a 49% and 51% relative decrease in pain levels in the resting position of the mandible and during mastication, respectively [47]. However, as there is a dearth of evidence pertaining to the specifics on the most efficacious utilization of LLLT for orthodontic pain reduction (e.g., energy/dose, application mode, frequency, time), the present clinical study aims to evaluate the efficacy of LLLT vs. placebo, following orthodontic ES placement in adult patients treated with fixed orthodontic appliances, introducing new variables (i.e., energy levels, regimes) not assessed in our earlier placebo-controlled study.

## 2. Materials and Methods

### 2.1. Study Setting, Inclusion and Exclusion Criteria, Determination of the Sample Size

Participants of the study were included from the adult patient pool at the Department of Orthodontics and Pediatric Dentistry (DOPD), Faculty of Dentistry, University of Szeged, who were recommended orthodontic treatment. The study was carried out between 26 November 2021 and 31 March 2022. Similar to our earlier study [47], participants were included based on the following criteria: *(a)* individuals between 18 and 50 years of age, *(b)* presenting with completely erupted second molars, without open interproximal contacts of the first molars, *(c)* good overall health, without the existence of known systemic disorders, *(d)* they were caries-free without gingivitis or any other periodontal problems, *(e)* no previous orthodontic treatment in the medical history, *(f)* willing to participate in the study. On the other hand, individuals were excluded if: *(a)* they failed to meet the general inclusion criteria or were unwilling to take part, *(b)* prior oral LLLT treatment in the medical history, *(c)* use of NSAIDs, other analgesic drugs or local anesthetics for pain management 6 weeks before the initiation of orthodontic treatment, *(d)* consumption of any type of tobacco products, *(e)* pregnant of lactating women.

Based on earlier published evidence, and our own experiences in the previous RCT [47], mean (± standard deviations; SD) pain readings of 25.0 ± 7.0, and a mean reduction in pain by 30% due to LLLT were expected to be detected on the visual analog scale (VAS) during the study, which were considered during the sample size determination process. Sample size calculations were carried out using the ClinCalc platform [48], where the required sample size of *n* = 32 was determined, considering the four-quadrant (three different LLLT treatment regimens and placebo), split-mouth study design, an alpha (α) set at 0.05, a beta (β) set at 0.2 and statistical power set at 0.8 (80%).

### 2.2. Ethical Considerations, Informed Consent

The study was conducted in accordance with the Declaration of Helsinki (1975, latest revision: 2024 [49]) and national and institutional ethical standards. Ethical approval for the study protocol was obtained from the National Institute of Pharmacy and Nutrition, Department of Health Technology, Hungary (reference number: OGYÉI/38078-6/2020; approval date: 3 July 2020). Furthermore, the Human Institutional and Regional Biomedical Research Ethics Committee, University of Szeged, was informed about the above-mentioned ethical approval and registered the study locally (reference number: 190/2020-SZTE; 14 December 2020).

Before participating in the study, participants were informed about the aims of the research, the privacy and confidentiality of their data, and that their participation in the research is voluntary, and they may withdraw from the study at any time without any consequences to their medical care. Written informed consent was obtained from patients agreed to participate in the study. No incentives (i.e., monetary, gifts or other renumeration) were provided to take part in the study.

### 2.3. Study Design

To avoid inter-individual variability between participants from confounding our results, a randomly assigned, split-mouth design was used [50]. Patients’ upper and lower dental arches were divided into right and left halves (i.e., quadrants). In each participant, three quadrants were used to administer the different LLLT treatment regimens, with the fourth quadrant designated as the placebo. The random assignment of quadrants to receive LLLT or placebo was determined using cards denoting the possible four quadrants and the four treatment variations, respectively [51]. Participants were asked to blindly choose from prepared cards to determine assignment; to ensure double-blinding, patients and the operator were blinded to the assignment of quadrants and treatments. Participants were asked not to use NSAIDs, other analgesic drugs, or local anesthetics during the study period.

### 2.4. Application Protocol, Treatment Regimens

ES placement (Dentalastic^®®^ separators, blue, Dentaurum^®®^, Ispringe, Germany) was carried out for all patients on the first permanent molars (mesially and distally) in both quadrants of lower, as well as upper jaws, as previously described [47]. The efficacy of LLLT was evaluated in two energy levels (i.e., intensity; 6 and 12 J, respectively) and regimes (continuous vs. pulsed). The assigned LLLT treatments in the appropriate three quadrants were applied as follows: regime 1 (R1: 6 J, continuous), regime 2 (R2: 12 J, continuous), regime 3 (R3: 6 J, pulsed), while placebo treatment (P) was applied in the remaining, fourth quadrant.

### 2.5. Laser Irradiation

A low-level medical gallium-aluminum-arsenide (GaAlAs) diode laser device (Medency Primo dental laser, Vicenza, Italy; wavelength: 980 nm, power: 0.1–0.2 W, producing 6–12 J energy), with either a continuous or pulsed wave mode on the first permanent molars in the appropriate treatment (R1–R3) quadrants. As previously [47], specified points in each quadrant were treated from mesial, central, and distal directions on the mucosa, both buccally and lingually/palatinally, with 10–10 s exposures for each, altogether for 60 s, producing either 36 or 72 J of energy per molar. During exposure, the laser tip was placed perpendicular to the long axis of the teeth, with close contact between the tip and the mucosa. Using the split-mouth design, and separation of the quadrants, patient received their LLLT treatment dose immediately after the placement of ESs.

During the treatment in the P quadrant, a similar procedure to that of R1–R3 was carried out, without actual laser irradiation being performed. To maintain blindedness, and to control for the participants’ pain-related behaviors, the handpiece was held the same way by the dentist on the placebo treatment side without laser irradiation, following a similar application protocol. To maintain bio-safety standards and to ensure blindness at the time of LLLT and placebo treatments, both the patient and the dentist (operator) wore goggles (safety glasses) designed to block the wavelength of the laser (980 nm), in addition to using earplugs to block out the typical voice of laser activity. As a result, neither the patient nor the operator could identify the type of therapy (i.e., LLLT vs. P) being administered in the specific quadrant; this was only known to the third person (technical staff) handling the equipment (turning laser irradiation on/off, and managing the different treatment regimes) at the time administering treatment [23]. To avoid the introduction of bias through avoiding intra-operator variations, a single operator placed the elastic separators and applied the laser and placebo treatment for all patients.

### 2.6. Determination and Registration of Pain Levels

Participants were assigned a code number on a separate data collection sheet, including a serial number, sex, date of birth (age), date of introduction to ESs, treatment regimens, and assigned quadrants, to ensure consistent evaluation and analysis. Pain levels were registered using a self-administered, standardized questionnaire. After receiving standardized instructions on how to score pain, participants were instructed to note their experienced pain intensity, according to localization (upper/lower jaw; right/left side) and the given time points, i.e., at 0 h (i.e., within 5 min of ES placement), 6 h (±30 min), 24 h (±30 min), 48 h (±30 min), and 72 h (±30 min) after application of the allocated treatment (laser/placebo), in resting position of mandible (corresponding to spontaneous pain) and during mastication (corresponding to pain on chewing) respectively, using a Visual Analog Scale (VAS), from 0 (no pain) to 100 (severe pain) [52,53,54,55]. The pain level reported by participants (i.e., the distance from zero to the mark) was measured using a digital caliper (MIB Messzeuge GmbH, Spangenberg, Germany). Before measurements, the digital calipers were calibrated daily, according to the manufacturer’s instructions.

### 2.7. Statistical Analysis

Data collected during the analysis were entered into spreadsheets (Microsoft Excel; Microsoft Corp., Redmond, WA, USA) for data management, which were later transferred to Statistical Package for Social Sciences v.22.0 (SPSS; IBM Corp., Endicott, NY, USA) for analysis. Descriptive analysis included frequencies (*n*) and percentages (%) for categorical variables, and means ± SD or means ± SEM for continuous variables (i.e., participant age, pain level experienced on VAS). For continuous variables, normality testing was performed using Shapiro–Wilk tests and Q-Q (quantile-quantile) diagrams. To compare the efficacy of different treatment regimens, or pain levels at different time points, Welch’s one-way analysis of variance (Welch-ANOVA) was used with Games–Howell post-hoc tests, while comparisons on the basis of sex were performed via Welch t-tests, respectively. *p*-values below 0.05 (*p* < 0.05) were considered statistically significant. Missing data were not relevant during analyses.

### 2.8. Reporting Guidelines, Trial Registration

The study has been registered on clinicaltrials.gov, under the identifier ID NCT07456709 (https://clinicaltrials.gov/study/NCT07456709, accessed on: 11 March 2026) “Efficacy of Different Regimens of 980 nm Low-Level Laser Therapy to Reduce Pain Caused by Orthodontic Separators: A Randomized, Double-Blind, Split-Mouth Placebo-Controlled Study (SZTEDENTlaser2) (SZTE-DENT-2021-38078-6)” (date of registration: 2 March 2026, retrospectively registered). Reporting of our study adheres to the guidelines set by the Consolidated Standards of Reporting Trials (CONSORT) statement [56].

## 3. Results

### 3.1. Characteristics of the Participants

**Figure 1:**
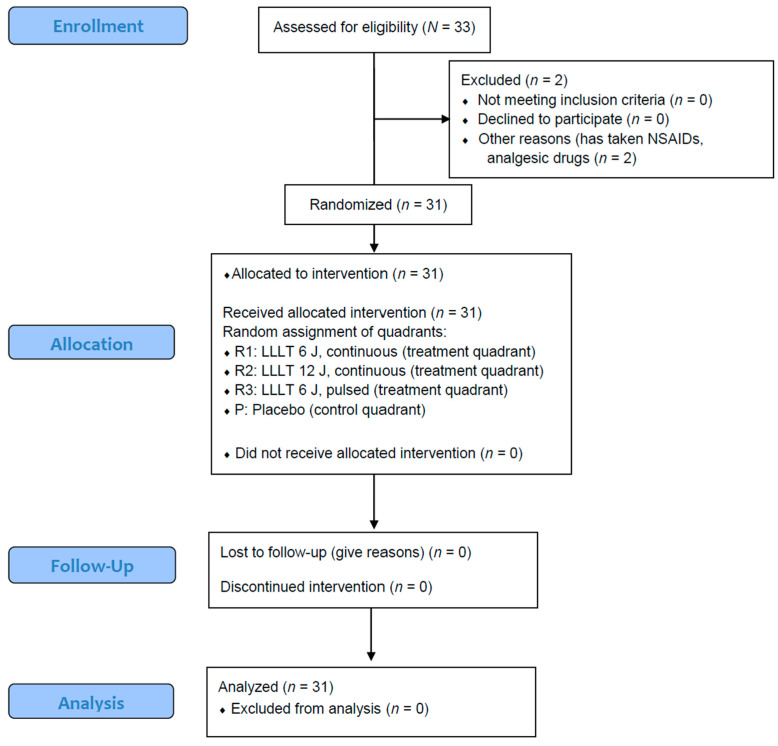
CONSORT flowchart describing the participants eligible (*N* = 33) and the final net number of patients (*n* = 31) enrolled and analyzed in the study; LLLT—low-level laser therapy, R1–3—treatment regimens 1–3, P—placebo control, NSAID—non-steroidal anti-inflammatory drugs.

A convenience sample of consecutive adult patients attending the DOPD were invited to participate in our study; those willing were screened for eligibility, according to our inclusion criteria. This approach ensured that the study population was representative of the typical adult clinical demographic treated at our institution; no external recruitment was conducted. The net sample size of the study initially involved *n* = 33 volunteers; however, *n* = 2 individuals (1-1 male and female, respectively) were excluded on the basis of consuming NSAIDs, other analgesic drugs, or local anesthetics following ES placement. Thus, *n* = 31 volunteers were included in the study, who provided the necessary information and informed consent to participate, and have fully documented their experienced pain intensity levels, according to instructions (as this is below the designated sample size, post-hoc power analysis revealed statistical power at 0.79 (79%)). The sample included 18 (58.1%) males and 13 (41.9%) females, aged between 19 and 32 years (mean ± SD: 23.43 ± 2.79). Participating in the study did not result in the interruption of the patients’ treatment, and none (*n* = 0) of the other participants used analgesic medications during the study period. None of the patients (*n* = 0) reported adverse effects, harms, or unintended consequences corresponding to the treatments. The CONSORT flowchart—representing the participants of the study—is shown in Figure 1.

### 3.2. Comparison of Pain Experience Between LLLT-Treated and Placebo Quadrants, Comparison According to Participants’ Sex

The pain experience reported by participants associated with the LLLT treatment regimens (R1–R3) and controls, corresponding to pain in the resting mandibular position and during mastication, is summarized in Table 1 and Table 2, respectively. At the first measurement point (i.e., directly after placing the ESs), no notable differences were shown among LLLT-treated and placebo quadrants, either in resting position (*p* = 0.877) or during mastication (*p* = 0.795). The highest mean VAS scores were observed for R2, followed by P, R1, and R3, indicating that continuous or pulsed irradiation with 6 J was the most effective (Table 1 and Table 2).

Similar observations were noted at the 6 h time point, where no notable differences were shown among LLLT-treated and placebo quadrants, either in resting position (*p* = 0.616) or during mastication (*p* = 0.353). In this case, pain readings were highest for R2, while lowest at R3 quadrants in resting position, and at R1 during mastication, respectively (Table 1 and Table 2). After 24 h, significant differences were detected between the pain readings of LLLT-treated and placebo quadrants, both in resting position (*p* = 0.046) and during chewing (*p* = 0.034); post-hoc analyses revealed differences between the R1 vs. R2 (*p* = 0.037) and R2 vs. R3 (*p* = 0.035) quadrants in resting position, and between the R1 vs. R2 (*p* = 0.025) and R1 vs. P (*p* = 0.016) quadrants during mastication, respectively. As earlier, pain readings were highest for R2, while lowest for R3 quadrants in resting position, and at R1 during mastication, respectively (Table 1 and Table 2).

Significant differences in pain experience persisted after 48 h, both measured in the resting position of the mandible (*p* = 0.005) and during mastication (*p* = 0.002). Subgroup comparisons showed significant differences between R1 vs. R2 (*p* = 0.002), R2 vs. R3 (*p* = 0.008), and R2 vs. P (*p* = 0.005) quadrants in the resting position of the mandible, while also between R1 vs. R2 (*p* = 0.006), R1 vs. R3 (*p* = 0.009) and R1 vs. P (*p* < 0.001) during mastication, respectively (Table 1 and Table 2). Pain readings were highest for R2, while lowest at R1 quadrants, both in resting position, and during mastication.

Finally, at 72 h, significant differences were shown in pain experience between the LLLT-treatment and placebo-control quadrants, in resting position (*p* = 0.011) and during mastication (*p* = 0.002), respectively; post-hoc tests revealed significant differences between the R1 vs. R2 (*p* = 0.005), R2 vs. R3 (*p* = 0.005), and R2 vs. P (*p* = 0.018) quadrants in resting position, while between R1 vs. R2 (*p* = 0.003), R1 vs. R3 (*p* = 0.014) and R1 vs. P (*p* < 0.001) during chewing, respectively (Table 1 and Table 2). At the final pain reading, mean VAS values were highest for R2, while lowest for R3 in the resting position, and for R1 during mastication.

When analyzed on the basis of the participants’ sex, no relevant differences were shown in reported pain experience among females vs. males, either in resting position of the mandible (mean ± SEM: 0 h: 4.49 ± 1.15 vs. 6.08 ± 1.21; *p* = 0.622; 6 h: 8.78 ± 1.54 vs. 15.79 ± 2.94; *p* = 0.508; 24 h: 16.49 ± 3.10 vs. 20.76 ± 3.08; *p* = 0.349; 48 h: 16.95 ± 2.69 vs. 20.37 ± 3.07; *p* = 0.729; 72 h: 18.19 ± 3.12 vs. 19.71 ± 3.03; *p* = 0.905) or during mastication (mean ± SEM: 0 h: 7.57 ± 1.84 vs. 9.29 ± 1.68; *p* = 0.376; 6 h: 13.46 ± 1.85 vs. 22.62 ± 3.30; *p* = 0.727; 24 h: 29.35 ± 3.87 vs. 34.57 ± 3.39; *p* = 0.371; 48 h: 36.00 ± 3.56 vs. 39.41 ± 3.40; *p* = 0.671; 72 h: 36.47 ± 4.39 vs. 40.99 ± 3.31; *p* = 0.422), measured at either time points, respectively.

### 3.3. Comparison of Pain Experience at Different Time Points During the Study

Significant differences in pain experience were identified over the time course of the study, in the case of all treatment regimens, either at resting position (R1: *p* = 0.034, R2: *p* < 0.001, R3: *p* = 0.002, P: *p* = 0.003) or during mastication (R1: *p* = 0.009, R2: *p* < 0.001, R3: *p* = 0.002, P: *p* < 0.001), with reported pain levels consistently increasing with the passage of time relative to the introduction of the ESs (Table 1 and Table 2).

When stratified by treatment regime, in resting position, there were differences in pain experience between measurements at 0 h vs. 24 h (*p* = 0.012), 0 h vs. 48 h (*p* = 0.009), and 0 h vs. 72 h (*p* = 0.007), while during chewing the same was observed at 0 h vs. 24 h (*p* = 0.002), 0 h vs. 48 h (*p* = 0.004), and 0 h vs. 72 h (*p* = 0.007), with regards to R1, respectively.

In case of R2, notable differences were seen between pain levels at 0 h vs. 24 h (*p* < 0.001), 0 h vs. 48 h (*p* < 0.001), 0 h vs. 72 h (*p* < 0.001), 6 h vs. 48 h (*p* = 0.003) and 6 h vs. 72 h (*p* = 0.005) in resting position of the mandible, and between pain levels at 0 h vs. 6 h (*p* = 0.024), 0 h vs. 24 h (*p* < 0.001), 0 h vs. 48 h (*p* < 0.001), 0 h vs. 72 h (*p* < 0.001), 6 h vs. 24 h (*p* = 0.032), 6 h vs. 48 h (*p* < 0.001) and 6 h vs. 72 h (*p* < 0.001) during mastication.

For R3, resting-position pain readings showed notable differences at 0 h vs. 24 h (*p* < 0.001), 0 h vs. 48 h (*p* < 0.001) and 0 h vs. 72 h (*p* < 0.001), while during mastication, similar differences were observed at 0 h vs. 24 h (*p* < 0.001), 0 h vs. 48 h (*p* < 0.001), 0 h vs. 72 h (*p* < 0.001), 6 h vs. 24 h (*p* = 0.025), 6 h vs. 48 h (*p* = 0.002), and 6 h vs. 72 h (*p* = 0.006), respectively.

Finally, in the placebo quadrants (P), considerable differences were seen in pain experience over time, i.e., between 0 h vs. 6 h (*p* < 0.001), 0 h vs. 24 h (*p* < 0.001), 0 h vs. 48 h (*p* < 0.001) and 0 h vs. 24 h (*p* < 0.001) in resting position, while between readings at 0 h vs. 6 h (*p* = 0.016), 0 h vs. 24 h (*p* < 0.001), 0 h vs. 48 h (*p* < 0.001), 0 h vs. 72 h (*p* < 0.001), 6 h vs. 24 h (*p* = 0.013), 0 h vs. 24 h (*p* < 0.001) and 0 h vs. 72 h (*p* < 0.001), respectively (Table 1 and Table 2).

The relative change in mean pain levels at the resting mandibular position and during mastication (compared with placebo quadrants) resulting from different treatments is summarized in Table 3. In the resting position, application of the R3 regime (6 J, pulsed mode) resulted in the greatest relative pain reduction (with values ~30% between 0–6 h), while during mastication, the R3 and R1 regime (6 J, continuous mode) had the most pronounced effect, with relative pain reduction tapering down (−33.10% → −15.52%) over time in case of R3, and becoming more pronounced over time (−32.22% → −54.52%) for R1, respectively. In contrast, change in pain levels was limited in the case of the R2 regime (12 J, continuous mode) during mastication, while pain readings were actually higher (+24.69% → +80.53%) compared to placebo, during the resting position of the mandible (Table 3).

## 4. Discussion

Pain experience is a common clinical feature during orthodontic treatments performed with fixed appliances, which could result in non-adherence to medical instructions and discontinuation of treatment [3,6,53,56,57]. Pain and discomfort may be described following a number of orthodontic interventions during the treatment process; however, it is most commonly associated with the use of ESs, the placement of archwires, canine retraction, and debonding procedures [23,27,28,29,30,31,32,33,34,35,39,58,59,60,61,62]. The initial phase of orthodontic treatment is characterized by the placement of ESs, to allow for the subsequent placement of the orthodontic bands, often associated with considerable amount of pain [23,27,28,29,30,31,32,33,34,35,39], associated with an inflammatory process, which alters blood flow after the application orthodontic mechanical forces, due to the relevance of numerous inflammatory mediators (prostaglandins, histamine serotonin, substance P) released from nerve endings [23,27,28,29,30,31,32,33,34,35,39,58,59,60,61,62]. As these compressive forces act on the periodontal ligaments, the inflammatory mediators induce ischemia, inflammation, and edema in the periodontal tissues [63,64,65]. Pain levels resulting from the abovementioned mechanisms cause considerable discomfort, reduce compliance and may lead to refusal of treatment in the affected patients [23,27,28,29,30,31,32,33,34,35,39,58,59,60,61,62]. Therefore, pain reduction and increasing patient comfort is paramount, especially in the initial phase of orthodontic treatment with fixed appliances.

For this purpose, NSAIDs and different types of anesthetic gels are commonly recommended, although there are other non-pharmacological treatment modalities, such as bite wafers, transcutaneous electrical nerve stimulation, and vibratory stimulation among the available pain management options [1,3,66,67]. In real-world practice, NSAIDs are some of the most commonly used and preferred choices by both orthodontists and patients for a considerable time, although their commonly occurring adverse effects are well-known [67]. NSAIDs exert their undesirable localized inhibitory effects on orthodontic tooth movement velocity via the COX-PGE2-RANKL axis: these drugs act by suppressing the cyclooxygenase-1 and 2 (COX-1, COX-2) enzymes (although studies have shown that selective COX-2 inhibitors, such as celecoxib, have even more pronounced effects), leading to decreased synthesis of prostaglandin E2 (PGE2) [67]. PGE2 acts as a key inflammatory mediator that plays a role in stimulating osteoclastogenesis and bone resorption: with reductions in PGE2, recruitment of osteoclasts to the pressure size of the periodontal ligament becomes limited, slowing down extracellular collagen remodeling activity (through the inhibition of matrix metalloproteinase activity, and reduced expression of the receptor activator of nuclear factor (NF)-κB ligand (RANKL) [67,68,69]. In addition to oral adverse effects in the oral cavity, the side effects associated with the extended systemic use of NSAIDs are also well-known [70,71].

Thus, non-pharmacological approaches, such as LLLT, have recently been suggested as a more pragmatic alternative for pain management during orthodontic treatment [27,28,29,30,31,32,33,34,35]. It has been described that the therapeutic benefits of LLLT are mediated through complex, interconnected cellular and molecular pathways, including activation of local microcirculation, as well as cellular metabolism (facilitating the activity of the mitochondrial respiratory chain, resulting in increased ATP production), the modulation of several transcription factors (such as NF-κB), stimulating the release of growth factors (e.g., tissue growth factor-beta), all leading to tissue remodeling and fibroblast proliferation [10,22,27]. Furthermore LLLT is also associated with intermittent, controlled releases of reactive oxygen species (ROS), also leading to tissue regeneration, remodeling and to the activation of anti-inflammatory signaling pathways [10,22,27,72]. However, the clinical efficacy of laser irradiation on biological systems may be influenced by many variables and parameters set during treatment [10,22,27]. From the patients’ safety perspective, the advantages of applying LLLT—when used according to the required safety standards—are clear over pharmacological interventions, as there are no known adverse events or contraindications related to their use. On the other hand, the effective application of LLLT in dentistry depends on several prerequisites: it is a professionally applied analgesic intervention, so attendance at the dental office/clinic is necessary (vs. self-medicating with NSAIDs), which may incur additional costs for patients. To use LLLT, dentists need to acquire appropriate instrumentation (and often staff and/or certification to use the device), which also incurs additional costs; while during LLLT treatments, strict adherence to safety standards and use of PPE is necessary [73]. Furthermore, an individuals’ perception of pain experience is subjective, and it is influenced by the patient’s individual pain threshold and responses [53]. Certain patient groups—whose adherence or tolerance to such treatment modalities may be limited—are also unable to benefit from LLLT-based therapies in dentistry.

To gain the greatest possible benefit from LLLT in routine clinical settings—and to clear out ambiguities related to its efficacy (vs. NSAIDs)—further studies are needed to determine the specific indications, patient populations, temporality during orthodontic treatment, and other technical details (e.g., intensity, laser operation mode, duration) for its use [22,23,31,34,41,42,43,44,45,46]. Available evidence pertaining to the efficacy of LLLT is contradictory: the systematic review and meta-analysis of Al-Hanbali et al. (2024) [30] assessed the effectiveness of pharmacological and non-pharmacological methods to reduce pain associated with ES placement, through systematic assessment of published RCTs (*n* = 31); their findings showed that both oral intake of NSAIDs (i.e., ibuprofen, paracetamol and naproxen) and LLLT were effective for pain relief at 6 h and 24 h after orthodontic separation, although the certainty of evidence was weak to moderate. In the systematic review and network meta-analysis by Gao et al. (2025), the analgesic efficacy of ibuprofen and LLLT was compared at 6 and 24 h after ES placement, based on the published data in 23 RCTs [74]. While compared to the control groups, both interventions resulted in significant pain reductions, ibuprofen resulted in a more immediate pain relief (i.e., 6 h), while LLLT presented its effects over the more sustained period of 24 h; their summary recommended a time-dependent, multimodal approach for orthodontic pain control in orthodontic practice, taking into account the severity of discomfort and individual, patient-specific factors during treatment selection [74].

The study of Owayda et al. [40] evaluated the analgesic efficacy of LLLT and paracetamol-caffeine in the management of orthodontic pain during different stages of the treatment; their findings indicated that, while LLLT considerably reduced pain levels during peak pain experience at the separation phase, it was less effective long-term (>24 h). In contrast, the paracetamol–caffeine combination was ineffective throughout the orthodontic treatment period, emphasizing that both LLLT and the drug combination are relevant only in the case of low levels of pain experience. The RCT reported by Golshah et al. (2025) assessed the analgesic efficacy of a single-dose 940 nm diode laser treatment (vs. placebo) after initial archwire placement, where LLLT did not provide significant reductions in the participants’ pain scores in the treatment arm [39]. Furthermore, the Bayesian network meta-analysis of 37 studies, performed by Dong et al. (2026)—with the aim of assessing the effectiveness of pharmacological and non-pharmacological interventions for post-orthodontic pain management—has concluded that compared to NSAIDs (especially naproxen and etoxicoxib) were the most effective, while describing the role of LLLT as adjunctive, to provide additional benefits [27].

Previous studies evaluating the efficacy of LLLT in reducing ES-placement-associated pain have applied numerous different LLLT treatment protocols and variables—including the device used, wavelength, energy applied, as well as the population involved, duration of the investigations, place and frequency of LLLT-application, outcome measurements—however, most of these studies only compared one type (frequency, intensity) of treatment in the intervention arm vs. the control, with the exception of two studies, to the best of our knowledge. AlSayed Hasan et al. (2018) [37] compared a single application of LLLT with 4 J or 16 J of energy (using a Ga-Al-As laser device at an 830 nm wavelength) vs. placebo treatment, where they could not confirm improvements in pain experience with LLLT. They concluded that LLLT—especially high-energy alternatives—were ineffective when used for analgesic effects. On the other hand, their study also emphasized that changing parameters like wavelength, application protocol, exposure time, or frequency of irradiation could lead to increased clinical efficacy. Furthermore, the study of Almallah et al. (2020) [46] compared the effectiveness of single and double doses (vs. placebo) of LLLT (4 J/m^2^ energy) in pain reduction associated with ES placement. Their study showed that both single- and double-dose LLLT have led to significant reductions in post-separation pain, while no statistical differences were detected between the two dosing regimes.

In our earlier study [47], the analgesic properties of a single continuous application of LLLT with 6 J energy were evaluated in a similar population at the DOPD, following ES placement; these results highlighted that LLLT administration may have beneficial effects on pain reduction, especially during mastication after 6–48 h following the separation procedure, based on self-reported pain experience (VAS scores). Given the disparities in the available literature on LLLT efficacy as an analgesic modality in orthodontics, our aim was to expand the scope of our studies to provide more clarity on the applicability of LLLT. The present study compared three treatment regimens (with variables including intensity and operation mode) with placebo, randomly assigned and applied to both dental arches; to the best of our knowledge, no similar investigations exist on the efficacy of LLLT in the published literature. Our results have highlighted that higher-intensity (12 J) LLLT treatment was on par with or often worse than placebo, when considering reported pain experience via VAS scores, which corresponds to the earlier observations of AlSayed Hasan et al. [37]. In addition, no relevant differences between R1–R3 vs. the placebo control were shown directly after ES placement and 6 h subsequently; these findings contrast our earlier observations were greatest benefits (i.e., relative pain reduction) were noted after 6 h of LLLT [47]. On the other hand, the efficacy of continuous and pulsed 6 J treatments were shown from the 24 h mark, both in resting position of the mandible and during mastication; based on earlier studies, the intensity of orthodontic pain plateaus around 24 h following ES placement [22,23,28,34,38,39,40,41,42,43,44], which was demonstrated among our participants as well—therefore underscoring the potential usefulness of LLLT as a pain management modality in the vulnerable period of orthodontic treatment.

Interestingly, in our sample of participants, the most effective pain reduction in the resting position was achieved using pulsed LLLT with 6 J energy in the majority of the registration appointments, while during mastication, the best analgesic effects were ensured by the continuously applied irradiation using 6 J energy. Overall, based on our observations, laser treatment with 6 J (either continuous or pulsed) might be preferable for reducing pain caused by elastomeric separators at the beginning of orthodontic treatment with fixed appliances—reinforcing our earlier RCT findings—while continuously applied 12 J energy was ineffective in this context. In our relatively age-homogeneous sample, sex did not affect reported pain experience; individual pain assessment during orthodontic treatment may be influenced by a plethora of factors—including age, gender and socio-cultural aspects—although previous studies have contested their relevance [2,3,4,7,8,75,76], supporting our findings. In addition to objective factors, such as applied wavelength, energy, treatment time and treatment repetition rate [23], the perceived clinical effectiveness of LLLT may also be determined by individual factors, such as emotional and psychological state, personal pain threshold and previous pain experience; taken together with the heterogeneity of the method and study designs used during the efficacy evaluations of LLLT so far, the literature available does not make the appropriate comparisons of effectiveness (i.e., LLLT vs. placebo, or LLLT vs. NSAIDs) straightforward [2,3,4,7,8,22,23,31,34,41,42,43,44,45,46,74,75,76].

### Limitations of the Study

The findings of the present study should be interpreted in light of its inherent limitations. First, this was a single-center investigation conducted at a tertiary-care clinical facility, which may have introduced self-selection bias (i.e., those who would be more proactive about their health anyway were included in our sample) and, consequently, limited the external validity and generalizability of the results. Furthermore, in dental epidemiology, clinic-based samples have been the standard for RCTs, but they must be distinguished from population-based samples. Second, although pain experience was assessed using a VAS scale—which has been extensively used in the literature and as a patient-reported outcome measure—this method is inherently subjective and is associated with known limitations in reliability and individual variability. A potential limitation of this study is its final sample size (*n* = 31), which, although similar to the sample populations often reported in other RCTs, yielded a post-hoc power of 0.79—slightly below the target power of 0.80 established during the initial study design. While this represents only a nominal deviation from the original protocol, it suggests a marginally increased risk of a Type II error (β); the study may have been slightly less sensitive in detecting very small effect sizes regarding orthodontic pain levels [VAS]. Nonetheless, given that the difference is minimal (1%), it is unlikely to substantively alter the clinical interpretations of the findings.

Differences observed between spontaneous pain and pain during mastication may have been influenced by participant-specific factors that could not be fully controlled for, even with the use of a split-mouth design. Such factors include variations in orthodontic anomalies (e.g., malocclusion type, crowding, or other quadrant-specific irregularities) that may have been present in one treated quadrant, but not in the contralateral side. Furthermore, the study population consisted exclusively of adult participants. Tissue characteristics and biological responsiveness to LLLT may differ substantially in pediatric populations, even among children of the same chronological age. These developmental differences could affect treatment outcomes and limit the applicability of the present findings to younger individuals. In addition, the compliance of children, their levels of dental treatment-related fear, and the influences of the parents, may all contribute to the experience associated with LLLT-based pain management in a pediatric population.

The present study utilized the split-mouth design; while this design is largely effective in controlling for inter-individual confounding variables—such as systemic health, pain threshold, and oral hygiene habits—the possibility or ‘carry-over’ effect between quadrants should be considered, mediated through the circulatory or nervous systems [77]. Nonetheless, the utilization of the highly localized application protocol was aimed at minimizing such potential interactions, and the distance between the experimental and control quadrants in this study also reduced the likelihood of direct light scattering or local biochemical diffusion influencing the contralateral side. Although the protocol for our trial was retrospectively registered, the primary and secondary outcomes reported in this manuscript remain strictly consistent with the original study protocol, which was designed prior to data collection and committed to preventing reporting bias. Finally, to elucidate the potential therapeutic effects of LLLT more comprehensively—and to potentially establish a dose–response relationship—future investigations should incorporate multiple laser systems and a range of energy parameters.

## 5. Conclusions

As pain experience is one of the principal reasons for limited patient compliance and abandoning orthodontic treatment (especially within the context of fixed appliances), assessment of effective, non-pharmacological modalities for pain management is critical to reach advantageous outcomes in both primary (clinical cure) and secondary (patient-reported outcomes, experiences) measures. Our current findings extend a previous RCT and reinforce our previous observations in adult patients requiring orthodontic treatment. Although these findings of our study are exploratory in nature (partly due to the sample size involved in our trial), the results suggest that a single application of LLLT—if used within specific perimeters—might be effective in reducing pain caused by orthodontic elastomeric separators, especially in the vulnerable period following separator placement—i.e., 24 h following separation—where pain experience plateaus in adults. Although the cautious interpretation of our results is warranted, indicating that future studies, relying on data from the use of LLLT in real-world routine conditions, are necessary to dependably establish the role, indications and place of LLLT in clinical practice, these underscore that LLLT might be a valuable addition to routine clinical practice in the future, helping to alleviate pain, while improving treatment outcomes and overall patient comfort, in the context of orthodontics.

## Figures and Tables

**Table 1:** Pain values measured on the visual analog scale in the quadrants treated by the different LLLT-regimens (R1–R3) and placebo (P), at the given time points for the resting position of the mandible (i.e., spontaneous pain).

	VAS Values (Mean ± SEM)					
LLLT Regimens	0 h	6 h	24 h	48 h	72 h	*p*-Values **
**R1**	4.86 ± 1.54	12.88 ± 3.93	17.96 ± 4.72	17.97 ± 4.45	18.27 ± 4.24	**0.034**
**R2**	7.27 ± 2.07	18.66 ± 4.52	29.73 ± 5.41	34.97 ± 4.69	35.38 ± 4.92	**<0.001**
**R3**	4.09 ± 1.22	10.01 ± 3.23	16.74 ± 4.20	19.06 ± 3.99	17.84 ± 4.16	**0.002**
**P**	5.83 ± 1.87	14.69 ± 3.98	20.99 ± 4.62	19.37 ± 4.35	20.78 ± 4.65	**0.003**
***p*** -values *	0.877	0.616	**0.046**	**0.005**	**0.011**	

LLLT—low-level laser therapy; VAS—visual analog scale, SEM—standard error of the mean * denoting *p*-values for comparisons between different treatment regimens at the same time points, ** denoting comparisons between different time points of the same treatment regimens, *p*-values < 0.05 were denoted in **boldface**.

**Table 2:** Pain values measured on the visual analog scale in the quadrants treated with the different LLLT regimens (R1–R3) and placebo (P), at the given time points, during mastication.

	VAS Values (Mean ± SEM)					
LLLT Regimens	0 h	6 h	24 h	48 h	72 h	*p*-Values **
**R1**	6.88 ± 2.11	13.83 ± 3.74	20.19 ± 4.28	19.33 ± 4.28	19.43 ± 4.61	**0.009**
**R2**	10.30 ± 3.02	20.80 ± 4.55	36.10 ± 5.52	39.90 ± 5.39	42.77 ± 5.78	**<0.001**
**R3**	6.79 ± 1.88	16.49 ± 3.99	27.69 ± 4.63	35.36 ± 4.84	36.09 ± 5.47	**0.002**
**P**	10.15 ± 1.88	20.49 ± 4.51	36.69 ± 5.32	42.17 ± 5.13	42.72 ± 5.07	**<0.001**
***p*** -values *	0.795	0.353	**0.034**	**0.002**	**0.002**	

LLLT—low-level laser therapy; VAS—visual analogue scale, SEM—standard error of the mean * denoting *p*-values for comparisons between different treatment regimens at the same time points, ** denoting comparisons between different time points of the same treatment regimens, *p*-values < 0.05 were denoted in **boldface**.

**Table 3:** Mean change in pain levels in the LLLT-treatment quadrants compared to the control (placebo) quadrants in the resting position of the mandible and during mastication (Δ%).

**Resting** **Position**	**0 h**	**6 h**	**24 h**	**48 h**	**72 h**
**R1**	**−16.64%**	**−12.32%**	**−14.43%**	**−7.22%**	**−12.08%**
**R2**	+24.69%	+27.02%	+41.64%	+80.53%	+70.26%
**R3**	**−29.85%**	**−31.86%**	**−20.24%**	**−1.60%**	**−14.15%**
**Mastication**	**0 h**	**6 h**	**24 h**	**48 h**	**72 h**
**R1**	**−32.22%**	**−32.50%**	**−44.97%**	**−54.17%**	**−54.52%**
**R2**	+1.48%	+1.51%	+1.61%	**−5.39%**	+0.12%
**R3**	**−33.10%**	**−19.53%**	**−24.52%**	**−16.15%**	**−15.52%**

Reductions in pain levels are denoted in **boldface**.

## Data Availability

The dataset is available from the Corresponding author on reasonable request.
